# Supplementary material for: A randomized pilot trial to evaluate the benefit of the concomitant use of atorvastatin and Raltegravir on immunological markers in protease-inhibitor-treated subjects living with HIV
Source: PLoS One. 2020 Sep 17;15(9):e0238575. doi: 10.1371/journal.pone.0238575 (PMC7498036; doi:10.1371/journal.pone.0238575)
Supplement: S1 Fig — (DOCX) [file pone.0238575.s001.docx]

**S1 Fig. Gating strategy for flow cytometry analysis.**
