# Supplementary material for: A randomized pilot trial to evaluate the benefit of the concomitant use of atorvastatin and Raltegravir on immunological markers in protease-inhibitor-treated subjects living with HIV
Source: PLoS One. 2020 Sep 17;15(9):e0238575. doi: 10.1371/journal.pone.0238575 (PMC7498036; doi:10.1371/journal.pone.0238575)
Supplement: S1 Table — (DOCX) [file pone.0238575.s002.docx]

**S1 Table: Changes in lipid profile**

|  | | Control group | | |  | | Raltegravir group | | | |
| --- | --- | --- | --- | --- | --- | --- | --- | --- | --- | --- |
|  | **Baseline** | | **W24** | **W72** | |  | | **Baseline** | **W24** | **W72** |
| Total cholesterol (mmol/L)  Mean (SD) | 4.9 (0.9) | | 4.7 (1.0) | 3.8 (0.9)^*^ | |  | | 4.7 (0.8) | 4.4 (0.7) | 3.7 (0.7)^*^ |
| LDL cholesterol (mmol/L)  Mean (SD) | 2.8 (0.8) | | 2.8 (0.8) | 1.9 (0.7)^*^ | |  | | 2.7 (0.7) | 2.7 (0.6) | 2.1 (0.6)^*^ |
| Triglycerides (mmol/L)  Mean (SD) | 1.8 (1.2) | | 1.4 (0.8) | 1.5 (0.7)^*^ | |  | | 2.0 (1.5) | 1.2 (0.5)^*^ | 1.1 (0.7)^*^ |

^*^ Significant differences from baseline. No significant differences were seen between groups at w72 in any lipid parameter.
